# A cocrystal of 3α-hy­droxy­tirucalla-8,24-dien-21-oic acid and 3β-fluoro­tirucalla-7,24-dien-21-oic acid (0.897:0.103)

**DOI:** 10.1107/S1600536811011159

**Published:** 2011-03-31

**Authors:** S. Yousuf, R. S. T. Kamdem, P. Wafo, B. T. Ngadjui, Hoong-Kun Fun

**Affiliations:** a H.E.J Research Institute of Chemistry, International Center for Chemical and Biological Sciences, University of Karachi, Karachi 75270, Pakistan; b Department of Chemistry, Higher Teachers Training College, University of Yaounde I, PO Box 48 Yaounde, Cameroon; c Department of Organic Chemistry, University of Yaounde I, PO Box 812 Yaounde, Cameroon; d X-ray Crystallography Unit, School of Physics, Universiti Sains Malaysia, 11800 USM, Penang, Malaysia

## Abstract

The title compound, 0.897C_30_H_48_O_3_.0.103C_30_H_47_O_2_F is a co-crystal of two triterpenes isolated from the resin of *Canarium schweinfurthiiand* Engl. Both triterpenes consists of four *trans-*fused rings having chair/half-chair/half-chair and envelope conformations. The mol­ecular conformations are stabilized by intra­molecular C—H⋯O hydrogen bonds, forming rings of *S*(7) graph-set motif. In the crystal, mol­ecules are linked by inter­molecular O—H⋯O and C—H⋯O inter­actions, forming sheets parallel to (001). All atoms. excepting the axially-oriented hydroxyl group in the major component and the equatorially-oriented fluorine atom in the minor component, are overlapping.

## Related literature

For the crystal structure of 3α-hy­droxy­tirucalla-7,24-diene-21-oic acid, see: Mora *et al.* (2001 ▶). For the crystal structure of 3α-hy­droxy­tirucalla-8,24-diene-21-oic acid, see: Yousuf *et al.* (2011 ▶). For the biological activity of *canarium schweinfurthiiand*, see: Atawodi (2010) ▶; Dongmo *et al.* (2010 ▶). For the stability of the temperature controller used in the data collection, see: Cosier & Glazer (1986 ▶).
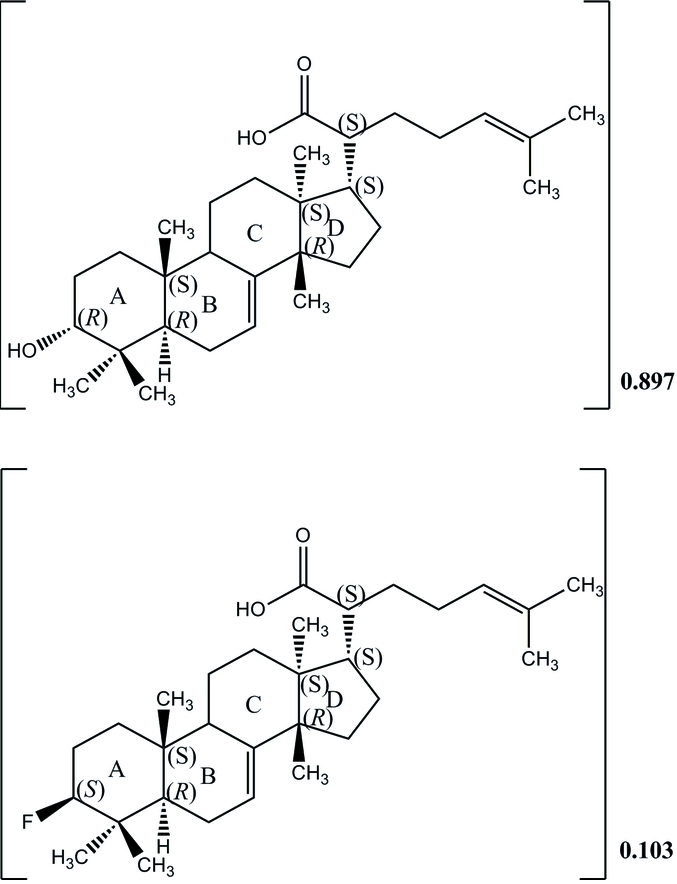


## Experimental

### 

#### Crystal data

0.897C_30_H_48_O_3_·0.103C_30_H_47_O_2_F
*M* *_r_* = 455.88
Trigonal, 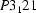

*a* = 11.2868 (9) Å
*c* = 36.446 (3) Å
*V* = 4020.9 (5) Å^3^
*Z* = 6
Mo *K*α radiation
μ = 0.07 mm^−1^
*T* = 100 K
0.29 × 0.24 × 0.13 mm

#### Data collection

Bruker SMART APEXII DUO CCD area-detector diffractometer
Absorption correction: multi-scan (*SADABS*; Bruker, 2009) ▶ *T*_min_ = 0.980, *T*_max_ = 0.991
27808 measured reflections
4454 independent reflections
4347 reflections with *I* > 2σ(*I*)
*R*_int_ = 0.105

#### Refinement

*R*[*F* ^2^ > 2σ(*F* ^2^)] = 0.058
*wR*(*F* ^2^) = 0.153
*S* = 1.18
4454 reflections
316 parameters
H-atom parameters constrained
Δρ_max_ = 0.39 e Å^−3^
Δρ_min_ = −0.33 e Å^−3^

### 

Data collection: *APEX2* (Bruker, 2009 ▶); cell refinement: *SAINT* (Bruker, 2009 ▶); data reduction: *SAINT*; program(s) used to solve structure: *SHELXS97* (Sheldrick, 2008 ▶); program(s) used to refine structure: *SHELXL97* (Sheldrick, 2008 ▶); molecular graphics: *SHELXTL* (Sheldrick, 2008 ▶); software used to prepare material for publication: *SHELXTL* and *PLATON* (Spek, 2009 ▶).

## Supplementary Material

Crystal structure: contains datablocks global, I. DOI: 10.1107/S1600536811011159/rz2572sup1.cif

Structure factors: contains datablocks I. DOI: 10.1107/S1600536811011159/rz2572Isup2.hkl

Additional supplementary materials:  crystallographic information; 3D view; checkCIF report

## Figures and Tables

**Table 1:** Hydrogen-bond geometry (Å, °)

*D*—H⋯*A*	*D*—H	H⋯*A*	*D*⋯*A*	*D*—H⋯*A*
O1—H1*O*1⋯O2^i^	0.87	1.81	2.654 (3)	165
O3—H3*A*⋯O2^ii^	0.84	2.04	2.818 (4)	154
C12—H12*B*⋯O1	0.99	2.56	3.262 (4)	128
C22—H22*A*⋯O3^iii^	0.99	2.40	3.300 (5)	151

Symmetry codes: (i) 
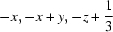
; (ii) 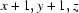; (iii) 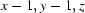.

**Fig. 1.**
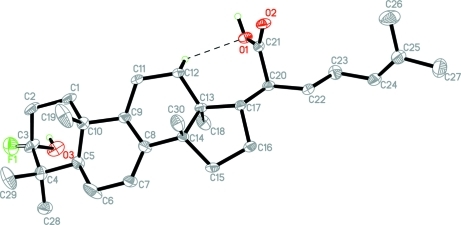
The molecular structure of the title compound showing 50% probability displacement ellipsoids. The intramolecular hydrogen bond is shown as a dashed line. Hydrogen atoms not involved in hydrogen bonding are omitted for clarity.

**Fig. 2.**
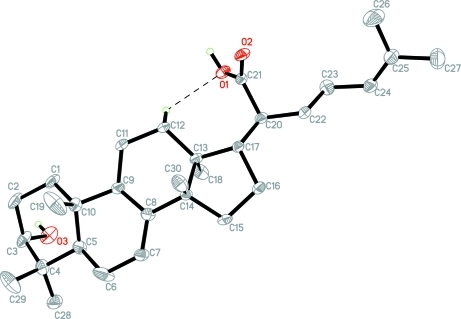
The molecular structure of the major component I, showing 50% probability displacement ellipsoids. The intramolecular hydrogen bond is shown as a dashed line. Hydrogen atoms not involved in hydrogen bonding are omitted for clarity.

**Fig. 3.**
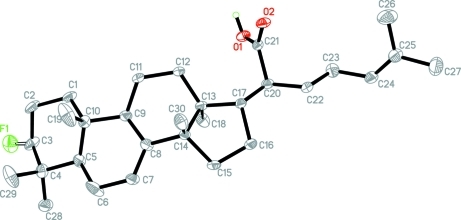
The molecular structure of the minor component II, showing 50% probability displacement ellipsoids. Hydrogen atoms are omitted for clarity.

**Fig. 4.**
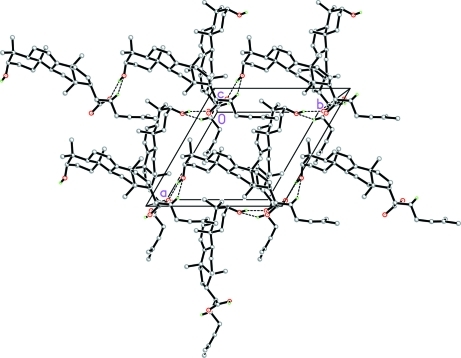
Crystal packing of the major component of the title compound, showing a two-dimensional molecular sheet parallel to the (001) plane. Only hydrogen atoms involved in hydrogen bonding (dashed lines) are shown.

## supplementary crystallographic information

### Comment

The title compound is a co-crystal of two triterpenes namely 3α-hydroxytirucalla-7,24-dien-21-oic acid (or epielemadienolic acid, I) as a major component (89.7%) and 3β-fluorotirucalla- 7,24-dien-21-oic acid (II) as minor component (10.3%). The co-crystal was isolated during the phytochemical investigation of the dichloromethane soluble part of the resins of the medicinally important plant *Canarium schweinfurthii* of Cameroon. The plant has been used for the treatment of a wide range of ailments including malaria, fever and diarrhea (Atawodi, 2010; Dongmo *et al.*, 2010). The refinement of the crystal structure revealed I and II as major (89.7%) and minor (10.3%) component, respectively with the difference that in II the axially oriented hydroxyl group attached to C3 has been replaced by the equatorially oriented fluorine atom. The asymmetric unit of the co-crystal (Fig. 1) consists of the mixture of I (Fig. 2) and II (Fig. 3). The crystal structure of the major component I has already been reported and the space group (P3_1_21) and cell parameters were found to be similar to those previously reported (Mora *et al.*. 2001, Yousuf *et al.*, 2011). However the minor component II was found to be a new triterpene. In both components the molecular structure showed that the *trans* fused rings A/B/C and D adopt chair [Q= 0.550 (4) Å, θ = 7.1 (4)° and φ = 88 (3)°] / half-chair [Q= 0.530 (4) Å, θ = 49.5 (4)° and φ = 323.5 (6)°] / half-chair [Q= 0.652 (4) Å, θ = 100.4 (4)° and φ = 83.8 (3)°] and envelope [Q= 0.483 (2)Å and φ = 10.7 (4)°] conformations respectively. The chair and envelop conformations of rings C and D are stabilized by C12—H12B···O1 intramolecular hydrogen bond. In the crystal structure, the molecules are linked to form two-dimensional molecular sheets *via* O3—H3A···O2, O1—H1O1···O2 and C22—H22A···O3 intermolecular hydrogen bonds (symmetry codes as in Table 1) and arranged parallel to the (001) plane (Fig.2).The absolute configuration was assigned on the basis of our recently published triterpene crystal data (Yousuf *et al.*, 2011).

### Experimental

The resin (100 g) of *Canarium schweinfurthii* Engl. was collected in Yaounde, Cameroon, in May 2010 and identified by Professor Noumi, a botanist at the Department of Biology, University of Yaounde-1. A voucher specimen (HNC 25918.) was deposited at the National Herbarium of Cameroon in Yaounde. The resin (100 g) of *C. schweinfurthii* was allowed to dry under shade and extracted with dichloromethane. The extract (70 g) was subjected to column chromatography over silica gel (300 g, 60 × 5 cm) eluting with hexane followed by a mixture of n-hexane–EtOAc in order to increase polarity. The fractions eluted were monitored by thin layer chromatography and similar fractions were combined to give seven fraction FrA-FrG. Fraction FrA (200 mg), obtained on elution with a mixture of n-hexane-EtOAc (8:2 *v*/*v*), was subjected to further column chromatography over silica gel (70 g, 60 cm^3^ × 3, hexane-acetone equimolar solution) to yield crystals of the title compound. Recrystallization from n-hexane gave colourless crystals (60 mg).

### Refinement

H atoms on the C of methyl, methylene, methine and oxygen were positioned geomatrically with C–H = 0.98–1.00 Å and O–H = 0.86 Å, respectively and constrained to ride on their parent atoms with *U*_iso_(H) = 1.2*U*_eq_(CH_2_, CH) and 1.5*U*_eq_(CH_3_, OH). A rotating group model was applied to the methyl groups. The crystal is a twin with twin law -1 0 0  0 - 1 0  0 0 1 and BASF = 0.1815 (16). Friedel pairs were merged in the last refinement cycles.

### Crystal data

**Table:**

0.897C_30_H_48_O_3_·0.103C_30_H_47_O_2_F	*D*_x_ = 1.130 Mg m^−^^3^
*M**_r_* = 455.88	Mo *K*α radiation, λ = 0.71073 Å
Trigonal, *P*3_1_21	Cell parameters from 10350 reflections
Hall symbol: p 31 2"	θ = 2.1–30.1°
*a* = 11.2868 (9) Å	µ = 0.07 mm^−^^1^
*c* = 36.446 (3) Å	*T* = 100 K
*V* = 4020.9 (5) Å^3^	Block, colourles
*Z* = 6	0.29 × 0.24 × 0.13 mm
*F*(000) = 1506	

### Data collection

**Table:**

Bruker SMART APEXII DUO CCD area-detector diffractometer	4454 independent reflections
Radiation source: fine-focus sealed tube	4347 reflections with *I* > 2σ(*I*)
graphite	*R*_int_ = 0.105
φ and ω scans	θ_max_ = 30.1°, θ_min_ = 2.1°
Absorption correction: multi-scan (*SADABS*; Bruker, 2009)	*h* = −15→15
*T*_min_ = 0.980, *T*_max_ = 0.991	*k* = −13→15
27808 measured reflections	*l* = −51→38

### Refinement

**Table:**

Refinement on *F*^2^	Primary atom site location: structure-invariant direct methods
Least-squares matrix: full	Secondary atom site location: difference Fourier map
*R*[*F*^2^ > 2σ(*F*^2^)] = 0.058	Hydrogen site location: inferred from neighbouring sites
*wR*(*F*^2^) = 0.153	H-atom parameters constrained
*S* = 1.18	*w* = 1/[σ^2^(*F*_o_^2^) + (0.0644*P*)^2^ + 1.6942*P*] where *P* = (*F*_o_^2^ + 2*F*_c_^2^)/3
4454 reflections	(Δ/σ)_max_ < 0.001
316 parameters	Δρ_max_ = 0.39 e Å^−^^3^
0 restraints	Δρ_min_ = −0.33 e Å^−^^3^

### Special details

**Table:**

Experimental. The crystal was placed in the cold stream of an Oxford Cryosystems Cobra open-flow nitrogen cryostat (Cosier & Glazer, 1986) operating at 100.0 (1) K.
Geometry. All e.s.d.'s (except the e.s.d. in the dihedral angle between two l.s. planes) are estimated using the full covariance matrix. The cell e.s.d.'s are taken into account individually in the estimation of e.s.d.'s in distances, angles and torsion angles; correlations between e.s.d.'s in cell parameters are only used when they are defined by crystal symmetry. An approximate (isotropic) treatment of cell e.s.d.'s is used for estimating e.s.d.'s involving l.s. planes.
Refinement. Refinement of *F*^2^ against ALL reflections. The weighted *R*-factor *wR* and goodness of fit *S* are based on *F*^2^, conventional *R*-factors *R* are based on *F*, with *F* set to zero for negative *F*^2^. The threshold expression of *F*^2^ > σ(*F*^2^) is used only for calculating *R*-factors(gt) *etc*. and is not relevant to the choice of reflections for refinement. *R*-factors based on *F*^2^ are statistically about twice as large as those based on *F*, and *R*- factors based on ALL data will be even larger.

### Fractional atomic coordinates and isotropic or equivalent isotropic displacement parameters (Å^2^)

**Table:**

	*x*	*y*	*z*	*U*_iso_*/*U*_eq_	Occ. (<1)
O1	0.1191 (2)	0.0008 (2)	0.12753 (5)	0.0221 (4)	
H1O1	0.1119	−0.0077	0.1511	0.033*	
O2	−0.1057 (2)	−0.0844 (2)	0.13357 (5)	0.0230 (4)	
O3	0.6534 (3)	0.8965 (3)	0.10496 (7)	0.0330 (8)	0.898 (8)
H3A	0.7204	0.9158	0.1187	0.049*	0.898 (8)
F1	0.693 (3)	1.048 (3)	0.1287 (7)	0.042 (7)	0.102 (8)
C1	0.4098 (3)	0.7220 (4)	0.14860 (7)	0.0268 (7)	
H1A	0.3651	0.6772	0.1720	0.032*	
H1B	0.4651	0.6811	0.1403	0.032*	
C2	0.5056 (4)	0.8752 (4)	0.15562 (8)	0.0335 (8)	
H2A	0.4524	0.9153	0.1660	0.040*	
H2B	0.5761	0.8875	0.1738	0.040*	
C3	0.5750 (3)	0.9496 (4)	0.12011 (8)	0.0278 (7)	
H3B	0.6369	1.0487	0.1257	0.033*	0.898 (8)
H3C	0.6079	0.8925	0.1103	0.033*	0.102 (8)
C4	0.4725 (3)	0.9373 (3)	0.09109 (9)	0.0249 (6)	
C5	0.3685 (3)	0.7821 (3)	0.08498 (8)	0.0241 (6)	
H5A	0.4249	0.7444	0.0746	0.029*	
C6	0.2630 (5)	0.7560 (4)	0.05513 (13)	0.0500 (13)	
H6A	0.2107	0.8014	0.0622	0.060*	
H6B	0.3116	0.7979	0.0319	0.060*	
C7	0.1654 (3)	0.6078 (3)	0.04875 (8)	0.0257 (6)	
H7A	0.1104	0.5813	0.0273	0.031*	
C8	0.1521 (3)	0.5079 (3)	0.07283 (8)	0.0217 (5)	
C9	0.2374 (4)	0.5422 (3)	0.10692 (8)	0.0299 (7)	
H9A	0.3193	0.5367	0.0989	0.036*	
C10	0.2987 (3)	0.6922 (3)	0.11976 (8)	0.0206 (5)	
C11	0.1746 (4)	0.4342 (3)	0.13671 (7)	0.0252 (6)	
H11A	0.1766	0.4589	0.1617	0.030*	
C12	0.1076 (4)	0.2838 (3)	0.12501 (7)	0.0277 (7)	
H12A	0.0164	0.2323	0.1367	0.033*	
H12B	0.1639	0.2453	0.1342	0.033*	
C13	0.0908 (3)	0.2624 (3)	0.08335 (7)	0.0180 (5)	
C14	0.0440 (3)	0.3596 (3)	0.06687 (7)	0.0176 (5)	
C15	0.0125 (4)	0.3087 (3)	0.02691 (8)	0.0273 (6)	
H15A	0.0967	0.3517	0.0119	0.033*	
H15B	−0.0549	0.3301	0.0159	0.033*	
C16	−0.0472 (3)	0.1517 (3)	0.02956 (7)	0.0237 (6)	
H16A	0.0009	0.1221	0.0124	0.028*	
H16B	−0.1458	0.1027	0.0234	0.028*	
C17	−0.0255 (3)	0.1210 (3)	0.06995 (7)	0.0180 (5)	
H17A	−0.1100	0.1000	0.0840	0.022*	
C18	0.2267 (3)	0.2887 (4)	0.06574 (10)	0.0294 (7)	
H18A	0.2646	0.2420	0.0801	0.044*	
H18B	0.2920	0.3872	0.0654	0.044*	
H18C	0.2094	0.2536	0.0406	0.044*	
C19	0.1860 (4)	0.7155 (4)	0.13545 (15)	0.0473 (11)	
H19A	0.1395	0.6511	0.1556	0.071*	
H19B	0.1197	0.7011	0.1161	0.071*	
H19C	0.2267	0.8094	0.1447	0.071*	
C20	−0.0046 (3)	−0.0042 (3)	0.07384 (7)	0.0191 (5)	
H20A	0.0854	0.0187	0.0627	0.023*	
C21	−0.0021 (3)	−0.0331 (3)	0.11447 (7)	0.0184 (5)	
C22	−0.1189 (3)	−0.1329 (3)	0.05494 (7)	0.0215 (5)	
H22A	−0.2073	−0.1581	0.0668	0.026*	
H22B	−0.1249	−0.1112	0.0289	0.026*	
C23	−0.0965 (4)	−0.2561 (4)	0.05649 (9)	0.0274 (6)	
H23A	−0.0041	−0.2285	0.0470	0.033*	
H23B	−0.1006	−0.2846	0.0824	0.033*	
C24	−0.2013 (4)	−0.3758 (4)	0.03450 (8)	0.0269 (6)	
H24A	−0.1861	−0.3718	0.0088	0.032*	
C25	−0.3113 (4)	−0.4852 (4)	0.04663 (8)	0.0288 (6)	
C26	−0.3528 (5)	−0.5105 (6)	0.08649 (10)	0.0518 (12)	
H26A	−0.2775	−0.4437	0.1018	0.078*	
H26B	−0.3741	−0.6031	0.0933	0.078*	
H26C	−0.4337	−0.5013	0.0903	0.078*	
C27	−0.4081 (5)	−0.5985 (4)	0.02111 (11)	0.0418 (9)	
H27A	−0.3788	−0.5722	−0.0043	0.063*	
H27B	−0.5010	−0.6138	0.0242	0.063*	
H27C	−0.4072	−0.6828	0.0269	0.063*	
C28	0.5494 (4)	0.9980 (4)	0.05503 (9)	0.0338 (8)	
H28A	0.6234	1.0919	0.0593	0.051*	
H28B	0.4860	0.9982	0.0367	0.051*	
H28C	0.5880	0.9425	0.0461	0.051*	
C29	0.4101 (4)	1.0252 (4)	0.10225 (15)	0.0462 (10)	
H29A	0.4819	1.1217	0.1028	0.069*	
H29B	0.3689	0.9975	0.1267	0.069*	
H29C	0.3397	1.0127	0.0844	0.069*	
C30	−0.0905 (3)	0.3400 (3)	0.08398 (10)	0.0267 (6)	
H30A	−0.1171	0.4003	0.0716	0.040*	
H30B	−0.0763	0.3627	0.1102	0.040*	
H30C	−0.1631	0.2446	0.0810	0.040*	

### Atomic displacement parameters (Å^2^)

**Table:**

	*U*^11^	*U*^22^	*U*^33^	*U*^12^	*U*^13^	*U*^23^
O1	0.0217 (10)	0.0320 (12)	0.0167 (7)	0.0165 (9)	0.0016 (8)	0.0012 (8)
O2	0.0198 (10)	0.0313 (11)	0.0140 (7)	0.0099 (9)	0.0026 (7)	0.0030 (8)
O3	0.0163 (12)	0.053 (2)	0.0305 (13)	0.0177 (13)	−0.0012 (10)	−0.0023 (13)
F1	0.040 (13)	0.043 (14)	0.053 (14)	0.029 (11)	−0.008 (11)	−0.003 (10)
C1	0.0229 (14)	0.0342 (17)	0.0131 (10)	0.0067 (13)	−0.0047 (10)	−0.0013 (11)
C2	0.0304 (16)	0.0350 (18)	0.0138 (11)	0.0003 (15)	−0.0012 (12)	−0.0055 (12)
C3	0.0149 (12)	0.0386 (18)	0.0187 (11)	0.0049 (13)	−0.0020 (11)	−0.0049 (12)
C4	0.0150 (12)	0.0214 (14)	0.0306 (14)	0.0033 (11)	−0.0048 (11)	−0.0039 (12)
C5	0.0181 (13)	0.0188 (13)	0.0281 (13)	0.0037 (11)	−0.0092 (11)	−0.0005 (11)
C6	0.042 (2)	0.0203 (16)	0.064 (3)	−0.0024 (15)	−0.035 (2)	0.0129 (17)
C7	0.0215 (13)	0.0218 (14)	0.0230 (12)	0.0027 (12)	−0.0109 (11)	0.0036 (12)
C8	0.0145 (12)	0.0195 (13)	0.0246 (12)	0.0036 (10)	−0.0066 (10)	0.0032 (11)
C9	0.0392 (18)	0.0205 (14)	0.0191 (11)	0.0068 (14)	−0.0115 (13)	0.0016 (11)
C10	0.0146 (12)	0.0222 (13)	0.0229 (11)	0.0078 (10)	−0.0029 (10)	−0.0015 (10)
C11	0.0350 (17)	0.0234 (13)	0.0114 (10)	0.0103 (13)	0.0001 (12)	0.0007 (10)
C12	0.045 (2)	0.0203 (13)	0.0131 (10)	0.0129 (14)	−0.0069 (13)	0.0013 (10)
C13	0.0182 (12)	0.0217 (13)	0.0133 (9)	0.0093 (11)	−0.0010 (9)	0.0026 (9)
C14	0.0158 (12)	0.0164 (12)	0.0139 (10)	0.0031 (10)	−0.0026 (9)	0.0020 (10)
C15	0.0366 (17)	0.0253 (14)	0.0160 (11)	0.0125 (14)	−0.0072 (12)	0.0045 (11)
C16	0.0257 (14)	0.0232 (14)	0.0157 (10)	0.0075 (12)	−0.0049 (11)	0.0005 (11)
C17	0.0177 (12)	0.0189 (12)	0.0128 (9)	0.0056 (10)	0.0007 (9)	0.0007 (9)
C18	0.0154 (13)	0.0244 (16)	0.0435 (17)	0.0062 (12)	0.0006 (13)	0.0051 (14)
C19	0.0253 (17)	0.0253 (17)	0.090 (3)	0.0118 (15)	0.022 (2)	0.010 (2)
C20	0.0190 (12)	0.0225 (13)	0.0137 (9)	0.0088 (11)	0.0023 (9)	0.0024 (10)
C21	0.0219 (13)	0.0179 (12)	0.0159 (9)	0.0104 (11)	0.0010 (10)	−0.0002 (9)
C22	0.0256 (14)	0.0227 (13)	0.0142 (9)	0.0107 (12)	−0.0007 (10)	−0.0005 (10)
C23	0.0264 (15)	0.0269 (16)	0.0289 (14)	0.0134 (13)	0.0018 (12)	−0.0012 (12)
C24	0.0357 (18)	0.0276 (15)	0.0185 (11)	0.0166 (14)	0.0020 (12)	−0.0010 (11)
C25	0.0286 (16)	0.0325 (16)	0.0245 (13)	0.0147 (14)	−0.0019 (12)	−0.0009 (12)
C26	0.033 (2)	0.068 (3)	0.0296 (16)	0.007 (2)	0.0078 (16)	0.0082 (19)
C27	0.040 (2)	0.036 (2)	0.0409 (18)	0.0125 (18)	−0.0050 (17)	−0.0098 (17)
C28	0.0324 (18)	0.0280 (16)	0.0263 (14)	0.0041 (14)	−0.0089 (13)	0.0011 (13)
C29	0.0299 (19)	0.0209 (16)	0.084 (3)	0.0097 (15)	0.005 (2)	0.0034 (19)
C30	0.0164 (13)	0.0207 (14)	0.0383 (16)	0.0059 (12)	0.0006 (12)	0.0005 (13)

### Geometric parameters (Å, °)

**Table:**

O1—C21	1.312 (4)	C14—C30	1.552 (4)
O1—H1O1	0.8651	C15—C16	1.552 (5)
O2—C21	1.229 (3)	C15—H15A	0.9900
O3—C3	1.406 (5)	C15—H15B	0.9900
O3—H3A	0.8400	C16—C17	1.559 (4)
O3—H3C	0.5288	C16—H16A	0.9900
F1—C3	1.27 (3)	C16—H16B	0.9900
C1—C2	1.534 (5)	C17—C20	1.551 (4)
C1—C10	1.539 (4)	C17—H17A	1.0000
C1—H1A	0.9900	C18—H18A	0.9800
C1—H1B	0.9900	C18—H18B	0.9800
C2—C3	1.529 (4)	C18—H18C	0.9800
C2—H2A	0.9900	C19—H19A	0.9800
C2—H2B	0.9900	C19—H19B	0.9800
C3—C4	1.521 (4)	C19—H19C	0.9800
C3—H3B	1.0000	C20—C21	1.519 (3)
C3—H3C	0.9601	C20—C22	1.541 (4)
C4—C29	1.532 (5)	C20—H20A	1.0000
C4—C28	1.535 (5)	C22—C23	1.533 (5)
C4—C5	1.562 (4)	C22—H22A	0.9900
C5—C6	1.528 (4)	C22—H22B	0.9900
C5—C10	1.568 (4)	C23—C24	1.506 (5)
C5—H5A	1.0000	C23—H23A	0.9900
C6—C7	1.491 (5)	C23—H23B	0.9900
C6—H6A	0.9900	C24—C25	1.314 (5)
C6—H6B	0.9900	C24—H24A	0.9500
C7—C8	1.376 (4)	C25—C26	1.509 (5)
C7—H7A	0.9500	C25—C27	1.516 (5)
C8—C9	1.499 (4)	C26—H26A	0.9800
C8—C14	1.516 (4)	C26—H26B	0.9800
C9—C11	1.517 (4)	C26—H26C	0.9800
C9—C10	1.547 (4)	C27—H27A	0.9800
C9—H9A	1.0000	C27—H27B	0.9800
C10—C19	1.533 (5)	C27—H27C	0.9800
C11—C12	1.534 (4)	C28—H28A	0.9800
C11—H11A	0.9500	C28—H28B	0.9800
C12—C13	1.534 (4)	C28—H28C	0.9800
C12—H12A	0.9900	C29—H29A	0.9800
C12—H12B	0.9900	C29—H29B	0.9800
C13—C18	1.548 (4)	C29—H29C	0.9800
C13—C17	1.554 (4)	C30—H30A	0.9800
C13—C14	1.556 (4)	C30—H30B	0.9800
C14—C15	1.540 (4)	C30—H30C	0.9800
			
C21—O1—H1O1	107.6	C14—C15—C16	104.7 (2)
C3—O3—H3A	109.5	C14—C15—H15A	110.8
C3—O3—H3C	26.1	C16—C15—H15A	110.8
H3A—O3—H3C	121.0	C14—C15—H15B	110.8
C2—C1—C10	113.4 (3)	C16—C15—H15B	110.8
C2—C1—H1A	108.9	H15A—C15—H15B	108.9
C10—C1—H1A	108.9	C15—C16—C17	106.6 (2)
C2—C1—H1B	108.9	C15—C16—H16A	110.4
C10—C1—H1B	108.9	C17—C16—H16A	110.4
H1A—C1—H1B	107.7	C15—C16—H16B	110.4
C3—C2—C1	110.9 (2)	C17—C16—H16B	110.4
C3—C2—H2A	109.5	H16A—C16—H16B	108.6
C1—C2—H2A	109.5	C20—C17—C13	118.1 (2)
C3—C2—H2B	109.5	C20—C17—C16	113.6 (2)
C1—C2—H2B	109.5	C13—C17—C16	102.4 (2)
H2A—C2—H2B	108.0	C20—C17—H17A	107.4
F1—C3—O3	82.0 (11)	C13—C17—H17A	107.4
F1—C3—C4	131.7 (11)	C16—C17—H17A	107.4
O3—C3—C4	107.4 (2)	C13—C18—H18A	109.5
F1—C3—C2	107.2 (11)	C13—C18—H18B	109.5
O3—C3—C2	110.9 (3)	H18A—C18—H18B	109.5
C4—C3—C2	112.4 (3)	C13—C18—H18C	109.5
F1—C3—H3B	30.0	H18A—C18—H18C	109.5
O3—C3—H3B	108.7	H18B—C18—H18C	109.5
C4—C3—H3B	108.7	C10—C19—H19A	109.5
C2—C3—H3B	108.7	C10—C19—H19B	109.5
F1—C3—H3C	95.1	H19A—C19—H19B	109.5
O3—C3—H3C	14.0	C10—C19—H19C	109.5
C4—C3—H3C	102.1	H19A—C19—H19C	109.5
C2—C3—H3C	102.2	H19B—C19—H19C	109.5
H3B—C3—H3C	122.6	C21—C20—C22	109.3 (2)
C3—C4—C29	109.3 (3)	C21—C20—C17	108.2 (2)
C3—C4—C28	108.7 (3)	C22—C20—C17	112.4 (2)
C29—C4—C28	106.2 (3)	C21—C20—H20A	109.0
C3—C4—C5	108.2 (3)	C22—C20—H20A	109.0
C29—C4—C5	115.7 (3)	C17—C20—H20A	109.0
C28—C4—C5	108.6 (3)	O2—C21—O1	122.7 (2)
C6—C5—C4	113.2 (3)	O2—C21—C20	122.5 (3)
C6—C5—C10	111.1 (3)	O1—C21—C20	114.8 (2)
C4—C5—C10	117.6 (2)	C23—C22—C20	113.5 (3)
C6—C5—H5A	104.5	C23—C22—H22A	108.9
C4—C5—H5A	104.5	C20—C22—H22A	108.9
C10—C5—H5A	104.5	C23—C22—H22B	108.9
C7—C6—C5	113.2 (3)	C20—C22—H22B	108.9
C7—C6—H6A	108.9	H22A—C22—H22B	107.7
C5—C6—H6A	108.9	C24—C23—C22	112.5 (3)
C7—C6—H6B	108.9	C24—C23—H23A	109.1
C5—C6—H6B	108.9	C22—C23—H23A	109.1
H6A—C6—H6B	107.7	C24—C23—H23B	109.1
C8—C7—C6	122.4 (3)	C22—C23—H23B	109.1
C8—C7—H7A	118.8	H23A—C23—H23B	107.8
C6—C7—H7A	118.8	C25—C24—C23	127.8 (3)
C7—C8—C9	121.6 (3)	C25—C24—H24A	116.1
C7—C8—C14	120.8 (2)	C23—C24—H24A	116.1
C9—C8—C14	117.5 (2)	C24—C25—C26	124.0 (3)
C8—C9—C11	113.8 (3)	C24—C25—C27	122.0 (3)
C8—C9—C10	114.3 (3)	C26—C25—C27	114.0 (3)
C11—C9—C10	115.9 (2)	C25—C26—H26A	109.5
C8—C9—H9A	103.6	C25—C26—H26B	109.5
C11—C9—H9A	103.6	H26A—C26—H26B	109.5
C10—C9—H9A	103.6	C25—C26—H26C	109.5
C19—C10—C1	111.3 (3)	H26A—C26—H26C	109.5
C19—C10—C9	110.2 (3)	H26B—C26—H26C	109.5
C1—C10—C9	108.4 (3)	C25—C27—H27A	109.5
C19—C10—C5	112.4 (3)	C25—C27—H27B	109.5
C1—C10—C5	108.7 (2)	H27A—C27—H27B	109.5
C9—C10—C5	105.6 (2)	C25—C27—H27C	109.5
C9—C11—C12	117.6 (2)	H27A—C27—H27C	109.5
C9—C11—H11A	121.2	H27B—C27—H27C	109.5
C12—C11—H11A	121.2	C4—C28—H28A	109.5
C11—C12—C13	113.8 (2)	C4—C28—H28B	109.5
C11—C12—H12A	108.8	H28A—C28—H28B	109.5
C13—C12—H12A	108.8	C4—C28—H28C	109.5
C11—C12—H12B	108.8	H28A—C28—H28C	109.5
C13—C12—H12B	108.8	H28B—C28—H28C	109.5
H12A—C12—H12B	107.7	C4—C29—H29A	109.5
C12—C13—C18	110.3 (3)	C4—C29—H29B	109.5
C12—C13—C17	116.6 (2)	H29A—C29—H29B	109.5
C18—C13—C17	108.3 (2)	C4—C29—H29C	109.5
C12—C13—C14	109.3 (2)	H29A—C29—H29C	109.5
C18—C13—C14	111.0 (2)	H29B—C29—H29C	109.5
C17—C13—C14	101.1 (2)	C14—C30—H30A	109.5
C8—C14—C15	117.1 (2)	C14—C30—H30B	109.5
C8—C14—C30	106.8 (3)	H30A—C30—H30B	109.5
C15—C14—C30	107.4 (3)	C14—C30—H30C	109.5
C8—C14—C13	110.7 (2)	H30A—C30—H30C	109.5
C15—C14—C13	101.5 (2)	H30B—C30—H30C	109.5
C30—C14—C13	113.4 (2)		
			
C10—C1—C2—C3	−57.4 (4)	C9—C11—C12—C13	−10.5 (5)
C1—C2—C3—F1	−148.0 (12)	C11—C12—C13—C18	81.3 (4)
C1—C2—C3—O3	−60.1 (4)	C11—C12—C13—C17	−154.6 (3)
C1—C2—C3—C4	60.2 (4)	C11—C12—C13—C14	−40.9 (4)
F1—C3—C4—C29	−70.9 (16)	C7—C8—C14—C15	35.8 (4)
O3—C3—C4—C29	−165.7 (3)	C9—C8—C14—C15	−147.8 (3)
C2—C3—C4—C29	72.0 (4)	C7—C8—C14—C30	−84.6 (4)
F1—C3—C4—C28	44.6 (16)	C9—C8—C14—C30	91.8 (3)
O3—C3—C4—C28	−50.3 (4)	C7—C8—C14—C13	151.4 (3)
C2—C3—C4—C28	−172.6 (3)	C9—C8—C14—C13	−32.2 (4)
F1—C3—C4—C5	162.4 (15)	C12—C13—C14—C8	63.1 (3)
O3—C3—C4—C5	67.5 (3)	C18—C13—C14—C8	−58.8 (3)
C2—C3—C4—C5	−54.8 (4)	C17—C13—C14—C8	−173.5 (2)
C3—C4—C5—C6	−177.7 (3)	C12—C13—C14—C15	−171.9 (3)
C29—C4—C5—C6	59.3 (5)	C18—C13—C14—C15	66.2 (3)
C28—C4—C5—C6	−59.9 (4)	C17—C13—C14—C15	−48.5 (3)
C3—C4—C5—C10	50.5 (4)	C12—C13—C14—C30	−57.0 (3)
C29—C4—C5—C10	−72.5 (4)	C18—C13—C14—C30	−178.9 (3)
C28—C4—C5—C10	168.3 (3)	C17—C13—C14—C30	66.4 (3)
C4—C5—C6—C7	178.8 (4)	C8—C14—C15—C16	156.0 (3)
C10—C5—C6—C7	−46.3 (5)	C30—C14—C15—C16	−83.9 (3)
C5—C6—C7—C8	14.2 (6)	C13—C14—C15—C16	35.4 (3)
C6—C7—C8—C9	−1.1 (6)	C14—C15—C16—C17	−9.2 (3)
C6—C7—C8—C14	175.2 (4)	C12—C13—C17—C20	−73.7 (3)
C7—C8—C9—C11	157.2 (3)	C18—C13—C17—C20	51.4 (3)
C14—C8—C9—C11	−19.2 (4)	C14—C13—C17—C20	168.0 (2)
C7—C8—C9—C10	21.0 (5)	C12—C13—C17—C16	160.6 (3)
C14—C8—C9—C10	−155.4 (3)	C18—C13—C17—C16	−74.3 (3)
C2—C1—C10—C19	−74.9 (3)	C14—C13—C17—C16	42.4 (3)
C2—C1—C10—C9	163.8 (3)	C15—C16—C17—C20	−149.2 (3)
C2—C1—C10—C5	49.5 (3)	C15—C16—C17—C13	−20.7 (3)
C8—C9—C10—C19	71.6 (4)	C13—C17—C20—C21	66.8 (3)
C11—C9—C10—C19	−63.7 (4)	C16—C17—C20—C21	−173.3 (2)
C8—C9—C10—C1	−166.4 (3)	C13—C17—C20—C22	−172.4 (2)
C11—C9—C10—C1	58.4 (4)	C16—C17—C20—C22	−52.5 (3)
C8—C9—C10—C5	−50.0 (4)	C22—C20—C21—O2	−49.3 (4)
C11—C9—C10—C5	174.7 (3)	C17—C20—C21—O2	73.4 (4)
C6—C5—C10—C19	−56.8 (4)	C22—C20—C21—O1	130.6 (3)
C4—C5—C10—C19	75.9 (4)	C17—C20—C21—O1	−106.7 (3)
C6—C5—C10—C1	179.5 (3)	C21—C20—C22—C23	−63.1 (3)
C4—C5—C10—C1	−47.7 (4)	C17—C20—C22—C23	176.8 (2)
C6—C5—C10—C9	63.4 (4)	C20—C22—C23—C24	−174.0 (2)
C4—C5—C10—C9	−163.9 (3)	C22—C23—C24—C25	−99.7 (4)
C8—C9—C11—C12	42.2 (5)	C23—C24—C25—C26	−0.2 (7)
C10—C9—C11—C12	177.7 (3)	C23—C24—C25—C27	−179.9 (4)

### Hydrogen-bond geometry (Å, °)

**Table:**

*D*—H···*A*	*D*—H	H···*A*	*D*···*A*	*D*—H···*A*
O1—H1O1···O2^i^	0.87	1.81	2.654 (3)	165
O3—H3A···O2^ii^	0.84	2.04	2.818 (4)	154
C12—H12B···O1	0.99	2.56	3.262 (4)	128
C22—H22A···O3^iii^	0.99	2.40	3.300 (5)	151

Symmetry codes: (i) −*x*, −*x*+*y*, −*z*+1/3; (ii) *x*+1, *y*+1, *z*; (iii) *x*−1, *y*−1, *z*.
